# Oriented feature pyramid network for small and dense wheat heads detection and counting

**DOI:** 10.1038/s41598-024-58638-y

**Published:** 2024-04-06

**Authors:** Junwei Yu, Weiwei Chen, Nan Liu, Chao Fan

**Affiliations:** 1 Key Laboratory of Grain Information Processing and Control (Henan University of Technology), Ministry of Education, Zhengzhou, 450001 China; 2 School of Artificial Intelligence and Big Data, Henan University of Technology, Zhengzhou, 450001 China; 3 College of Information Science and Engineering, Henan University of Technology, Zhengzhou, 450001 China; 4 Basis Department, PLA Information Engineering University, Zhengzhou, 450001 China

**Keywords:** Computer science, Information technology

## Abstract

Wheat head detection and counting using deep learning techniques has gained considerable attention in precision agriculture applications such as wheat growth monitoring, yield estimation, and resource allocation. However, the accurate detection of small and dense wheat heads remains challenging due to the inherent variations in their size, orientation, appearance, aspect ratios, density, and the complexity of imaging conditions. To address these challenges, we propose a novel approach called the Oriented Feature Pyramid Network (OFPN) that focuses on detecting rotated wheat heads by utilizing oriented bounding boxes. In order to facilitate the development and evaluation of our proposed method, we introduce a novel dataset named the Rotated Global Wheat Head Dataset (RGWHD). This dataset is constructed by manually annotating images from the Global Wheat Head Detection (GWHD) dataset with oriented bounding boxes. Furthermore, we incorporate a Path-aggregation and Balanced Feature Pyramid Network into our architecture to effectively extract both semantic and positional information from the input images. This is achieved by leveraging feature fusion techniques at multiple scales, enhancing the detection capabilities for small wheat heads. To improve the localization and detection accuracy of dense and overlapping wheat heads, we employ the Soft-NMS algorithm to filter the proposed bounding boxes. Experimental results indicate the superior performance of the OFPN model, achieving a remarkable mean average precision of 85.77% in oriented wheat head detection, surpassing six other state-of-the-art models. Moreover, we observe a substantial improvement in the accuracy of wheat head counting, with an accuracy of 93.97%. This represents an increase of 3.12% compared to the Faster R-CNN method. Both qualitative and quantitative results demonstrate the effectiveness of the proposed OFPN model in accurately localizing and counting wheat heads within various challenging scenarios.

## Introduction

Over the past 50 years, the global population has experienced unprecedented growth, posing a significant challenge in ensuring food security through increased yields of major cereals such as wheat, rice, and maize^1^. Wheat, as a staple in the human diet and a primary food source for domesticated animals worldwide, plays a critical role. Given the ongoing urbanization and upgrading of consumption patterns, it is predicted that a 60% increase in wheat production will be required by 2050. Recently, there has been a growing focus among scientists on studying wheat growth monitoring, health assessment, and plant breeding. In the breeding process, the number of wheat heads per unit area is a key trait that directly impacts yield potential^2^. However, accurately counting wheat heads in the wild is a labor-intensive and time-consuming task that still relies on manual observation. Therefore, the need for accurate detection and automated counting of wheat heads with new technologies has become crucial. With the rapid advancements in deep learning and computer vision, image detection based on artificial neural networks shows tremendous potential in providing fast, accurate, and cost-effective solutions for wheat head detection and counting.

The emergence of smartphones, unmanned aerial vehicles (UAV) equipped with affordable digital cameras has made in-field images more readily available. Consequently, several large and well-annotated wheat head datasets for wheat head detection and yield estimation have been proposed. Deep learning methods offer an alternative solution to the traditional manual measurement. Utilizing the Global Wheat Datasets, two worldwide competitions have been conducted: the Global Wheat Head Detection 2020 powered by Kaggle and the Global Wheat Challenge 2021 powered by AI crowd. These competitions attracted 2245 and 563 teams respectively, resulting in the development of more accurate and robust algorithms. The majority of these solutions rely on common computer vision methods for object detection, using horizontal bounding box annotation for each wheat head. Models developed for wheat head detection involve one-stage and two-stage detectors. One-stage detectors, such as YOLO (You Only Look Once)^3^ and SSD (Single Shot MultiBox Detector)^4^, directly predicts the bounding boxes and class probabilities of objects in a single pass of the input image. One-stage detectors tend to be faster and suitable for real-time applications, but they may sacrifice accuracy for small and overlapping objects. On the other hand, two-stage detector such as R-CNN and Faster R-CNN first generates a set of region proposals, then refines them with bounding box regression and classifies them into different categories using a convolutional neural network (CNN). Two-stage detectors typically achieve higher accuracy by leveraging more complex architectures and multi-stage processing pipeline but are slower in comparison.

**Figure 1:**
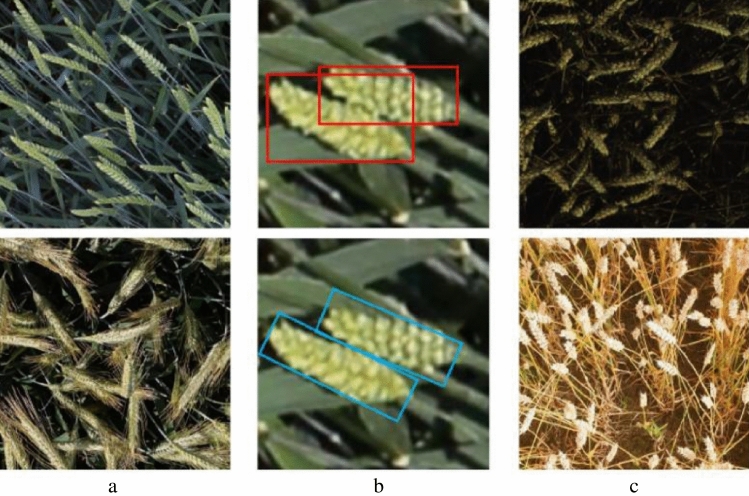
The challenging wheat scenarios due to diverse factors: (**a**) orientation by phototropism or wind, (**b**) boxes location and overlap of bounding boxes, the wheat targets are labeled with red horizontal and blue oriented boxes, respectively; (**c**) variations of variety, illumination and maturity.

With the active contributions of competition participants, significant progress has been made in wheat head detection using algorithms based on horizontal bounding boxes. However, there are still challenges in precise object representation and robust detection of wheat heads. These challenges are illustrated in Fig. 1 and arise due to multiple factors.

1. Orientation: Wheat heads tend to grow towards the sunlight or in a specific direction influenced by phototropism or natural wind. Additionally, the wind generated by UAVs during image acquisition can also impact the orientation of wheat heads. To accurately detect wheat heads, it is necessary to employ oriented object detection algorithms that can account for their orientation and enhance the accuracy of detection algorithms.
2. Aspect ratio and overlap: Wheat heads have a distinctive aspect ratio and often overlap with each other. They typically have a long and narrow shape, resulting in a high aspect ratio. Wheat heads often grow in dense clusters, causing them to overlap with each other. Figure 1b displays different annotations using horizontal bounding boxes and oriented bounding boxes. Traditional detection methods based on horizontal bounding boxes may struggle to accurately locate oriented wheat heads, leading to the inclusion of more background regions and imprecise representation of elongated wheat heads.
3. Variations: There are various variations in wheat varieties, illumination conditions, and maturity stages, all of which impact wheat head detection. Wheat varieties worldwide exhibit variations in shape, size, and color. Different illumination conditions, such as shadows, uneven illumination, or varying intensities, can make traditional detection methods less reliable. Additionally, the appearance of wheat heads is influenced by their maturity stages, introducing challenges for accurate detection and counting.

To address the aforementioned challenging issues, it is crucial to explore alternative methods for object detection that effectively address the complexities associated with wheat head detection. Notably, wheat heads in field images, as well as ships and vehicles in aerial images, exemplify small and densely packed rotated objects with a large aspect ratio. Drawing inspiration from oriented object detection in aerial images^5^. we advocate for the utilization of oriented bounding boxes in wheat head detection. This approach offers a more precise representation of objects by incorporating orientation information into the detection process, facilitating accurate identification and localization of wheat heads. By leveraging oriented object detection techniques, farmers and researchers can make informed decisions pertaining to crop management, yield estimation, and resource allocation.

Research has demonstrated that improving the size, diversity, and quality of the dataset proves more effective than increasing the complexity and depth of the network^6^. Given that the detection of wheat heads extends beyond a single region or country, it is imperative to develop a model capable of identifying them across various environments. In this study, we undertake the re-annotation of wheat images from the GWHD dataset^7^ by incorporating oriented bounding boxes. This approach allows for precise localization of the wheat heads while excluding unnecessary background or unrelated objects that might impede accurate wheat identification.

The main contributions are summarized in the following points:

1. A large-scale rotated wheat heads dataset RGWHD is introduced and the images are manually annotated using oriented bounding boxes(OBB). The RGWHD dataset provides a benchmark for small and dense rotated wheat heads detection.
2. We propsoe a novel wheat head detection model named the Oriented Feature Pyramid Network (OFPN). This model incorporates ResNeXt as the backbone for feature extraction, and utilizes Path-aggregation and Balanced Feature Pyramid Network (PBFPN) for effective fusion of multi-scale features. Additionally, the OFPN network employs a Soft-NMS algorithm to filter and refine proposal bounding boxes, improving detection performance in cases of overlapping wheat heads.
3. We conduct extensive experiments to compare our OFPN network with six state-of-the-art rotated object detection networks. Experimental results demonstrate the superiority of our proposed OFPN network in terms of rotated object detection accuracy, wheat category recognition, and wheat heads counting.

## Related work

### Object detection based on horizontal bounding boxes

Object detection is a fundamental task in computer vision that involves identifying and locating objects of interest within images. In addition to recognizing the presence of objects, accurate localization is achieved by marking their boundaries with horizontal bounding boxes. Object detection algorithms can be broadly classified into two types: one-stage^8^ and two-stage^9^. Two-stage algorithms, such as Faster R-CNN^10^, first generate a set of object candidates called object proposals using a dedicated proposal generator of Region Proposal Network (RPN)^11^. Subsequently, the classification and bounding box regression processes are performed. On the other hand, one-stage algorithms such as YOLO and SSD skip the intermediate step of generating object candidates. Instead, they directly employ a convolutional neural network to extract features for object classification and bounding box regression. Although one-stage algorithms are faster, they typically exhibit lower accuracy compared to two-stage algorithms.

Convolutional Neural Networks (CNNs) are deep learning algorithms specifically designed for image recognition and analysis. Their application has revolutionized the field of precision agriculture. Researchers have successfully utilized CNNs to automatically count and monitor wheat heads. And this process is crucial for estimating crop yield and making informed decisions about irrigation, fertilization, and harvesting^12^. Lu proposed TasselNet^13^, a deep convolutional neural network for accurately counting maize tassels in unconstrained field-based environments. TasselNet utilizes a local counts regression network architecture to address challenges such as in-field variations, resulting in excellent adaptability and high precision in maize tassel counting.

Fares Fourati^14^ developed a robust model that combines the Faster R-CNN and EfficientDet architectures, giving more prominence to the proposed final architectures and leveraging semi-supervised learning techniques to enhance previous models of objection detection. Fourati's approach was submitted in response to the Global Wheat Challenge on GWHD, and their method achieved a top 6% ranking in the competition. In order to address the limitation of labor-intensive data collection in wheat breeding, S. Khaki^15^ proposed a lightweight model WheatNet which utilizes a truncated MobileNetV2 and point-level annotations. WheatNet is robust and accurate in counting and localizing wheat heads across different environmental conditions.

M. Hasan^16^ proposed a region-based convolutional neural network model to accurately detect, count, and analyze wheat spikes for yield estimation subjected to three fertilizer treatments. They tested their approach on an annotated wheat dataset called SPIKE comprising 10 wheat varieties with images captured by high definition RGB cameras mounted on a land-based imaging platform. Wen^17^ utilized the GWHD dataset and introduced a novel wheat head detector named SpikeRetinaNet, which achieved outstanding detection performance.

Based on the GWHD dataset, the WheatLFANet model proposed by Ye, J^18^ is able to operate efficiently on low-end devices while maintaining high accuracy and utility. Jun S^19^ proposed a WHCnet model utilizing the Augmented Feature Pyramid Networks (AugFPN) to aggregate feature information and using cascaded Intersection over Union (IoU) threshold to remove negative samples to improve the training effect, and finally using a novel detection pipeline object counting method to count wheat sheaves from the top view in the field. Zhou, Q^20^ proposed the NWSwin Transformer to extract multiscale features and used a Wheat Intersection over Union loss by incorporating Euclidean distance, area overlapping, and aspect ratio, thereby leading to better detection accuracy. Wang, Y^21^ introduced the convolutional block attention module (CBAM) into the backbone network to make the model pay more attention to the target region of wheat ears and improve the detection results.

However, the solutions above were primarily based on horizontal bounding boxes, limiting their capabilities and robustness in detecting small and dense wheat heads with varying orientations.More importantly, they are not concerned about the fusion of feature information between non-adjacent feature maps, resulting in the loss of some feature information.

### Object detection based on oriented bounding boxes

In contrast to detection methods using horizontal bounding boxes, object detection techniques based on oriented bounding boxes offer advantages in terms of improved accuracy and better representation. Oriented object detection has numerous applications in computer vision and image analysis, proving valuable in various scenarios such as text detection and recognition, object detection in aerial imagery, autonomous driving, and medical imaging.

Oriented object detection algorithms can be categorized into anchor-based, which use a number of anchors with fixed scales and aspect ratios, and anchor-free, which are based on points. The anchor-based approach is utilized in the following models. Y. Jiang^22^ developed R2CNN for text detection, an innovative model based on Faster R-CNN. It extracts features using different pooled sizes while simultaneously predicting the text score, axis-aligned box, and inclined minimum area box. X. Xie^23^ proposed a novel RPN called oriented R-CNN, which generates high-quality oriented proposals rapidly while maintaining high detection accuracy and efficiency comparable to one-stage oriented detectors.

The models mentioned below are all based on anchor-free boxes. G. Cheng^24^ designed the Anchor-free Oriented Proposal Generator (AOPG), which generates oriented proposals instead of using sliding fixed-shape anchors on images. X. Wang^25^ developed PP-YOLOE-R, an efficient anchor-free rotated object detector. It incorporates several tricks to improve detection accuracy, including ProbIoU^26^ and a decoupled angle prediction head.

Zhonghua Li^27^ introduced FCOSR, an innovative rotated target detector that builds upon FCOS^28^ and utilizes a 2-dimensional Gaussian distribution to enable rapid and accurate prediction of objects. Drawing inspiration from object detection methods employing oriented bounding boxes, we anticipate that this approach will effectively address challenging aspects of wheat head detection, including the handling of oriented wheat heads and the overlap between predicted bounding boxes.

### Datasets

The available public datasets for wheat head detection include GWHD, SPIKE, ACID^29^, UWHD^30^, WED^31^. The GWHD dataset is a comprehensive collection of well-annotated wheat head images, compiled by nine research institutions across seven countries. It serves as a valuable resource for training robust models to accurately estimate the location and density of wheat heads in seven categories. The GWHD dataset can be accessed at https://www.kaggle.com/competitions/global-wheat-detection/data. The SPIKE dataset comprises 335 images captured at three distinct growth stages, covering ten different wheat varieties. The UWHD dataset consists of 550 images captured by a drone at an altitude of 10 m. The ACID dataset consists of 520 images taken in controlled greenhouse conditions, featuring 4158 labeled wheat heads with point annotations. The WED dataset contains 236 high resolution images with 30,729 wheat heads and derived the WEDU^32^ dataset with more accurate labeling information. For the specifics of each dataset, please refer to Table 1.

**Table 1:** Specifics of public wheat head datasets and the proposed RGWHD: Most of the existing public wheat datasets are mainly labeled with horizontal bounding boxing, and our proposed RGWHD dataset is labeled with oriented bounding boxes as the labeling information.

Dataset	Release	Environment	Resolution	Numbers	Instances	Annotation
GWHD	2020	Field	1024 × 1024	3422	188,445	Horizontal bounding box
SPIKE	2018	Lab	2001 × 1501	335	25,000	Horizontal bounding box
ACID	2017	Greenhouse	1956 × 1530	520	4158	Point
UWHD	2022	Field(UAV)	1120 × 1120	550	30,500	Horizontal bounding box
WEDU	2019	Field	6000 × 4000	236	30,729	Horizontal bounding box
RGWHD	2023	Field	1024 × 1024	700	25,677	Oriented bounding box

In order to address the challenges posed by oriented wheat heads, we constructed a new dataset named RGWHD based on GWHD, as the majority of existing datasets are labeled using horizontal bounding boxes. To achieve this, we randomly selected 100 images from seven categories within the GWHD dataset, resulting in a total of 700 images. RGWHD is comparable in size to most existing wheat head datasets. Given the complexity of image annotation, we relabeled the partially sampled images from GWHD, while leaving the remaining images for future research using weakly supervised learning. The roLabelImg annotation tool was utilized to annotate each image with oriented bounding boxes, using five parameters: (x, y, w, h, θ). Here, x and y represent the coordinates of the center point, w and h denote the width and height of the wheat head, and θ indicates the rotation angle of the bounding box.The constructed oriented bounding boxes labeled RGWHD was randomly divided into training, validation and test sets in the ratio of 8:2.The number of wheat ears included in the training-test dataset respectively is shown in Table 2.

**Table 2:** Details of training-test set division.

Category	Train	Test	Total
arvalis_1	3495	824	4319
arvalis_2	1650	389	2039
arvalis_3	2280	506	2786
ethz_1	5564	1469	7033
inrae_1	1693	371	2064
rres_1	3607	901	4508
usask_1	2314	614	2928
Total	20,603	5074	25,677

**Figure 2:**
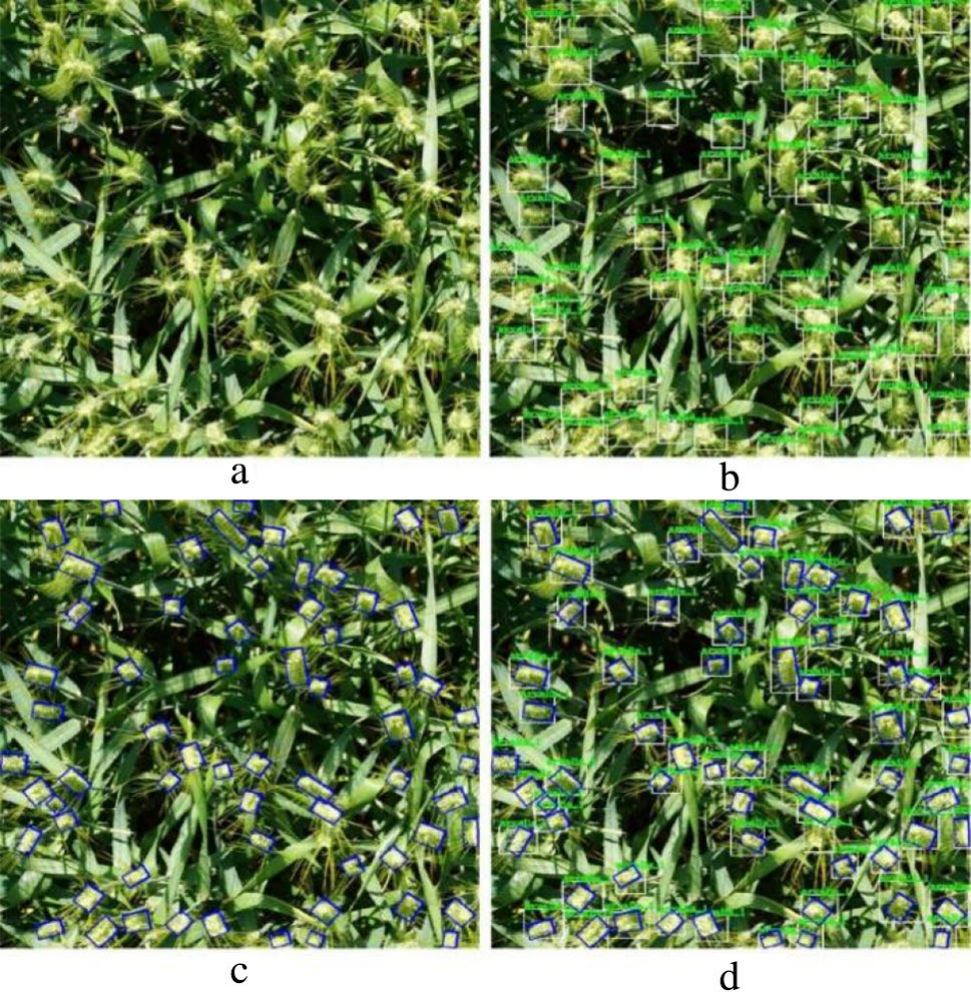
Visualization of the difference in area between horizontal and oriented bounding boxes: (**a**) original image, (**b**) annotated with horizontal bounding boxes, (**c**) annotated with oriented bounding boxes, (**d**) annotated with both horizontal and oriented bounding boxes.

The dataset proposed in this study, RGWHD, provides a more accurate representation of the wheat head by utilizing tighter bounding boxes. To assess this difference quantitatively, we compared the average area occupied by horizontal and oriented bounding boxes in all RGWHD images. The notable contrast in area between the two types of bounding boxes is presented in Table 2, while Fig. 2 visually illustrates the horizontal and oriented annotations. The average area is calculated using the following formula, providing a standardized measure for comparison.

1 $$A = \frac{{T_{a} }}{N}$$

where, $A$ represents the average area (in pixels) of each bounding box, $N$ is the number of bounding boxes, and $T_{a}$ is the total area of all the bounding boxes in this image.

The statistical results presented in Table 3 demonstrate a significant reduction in the average area occupied by the oriented bounding box (OBB) compared to the area occupied by the horizontal bounding box (HBB) across the seven wheat categories. Among these categories, the average relative proportion of area reduction reaches 49.51%, with arvalis_2 showing the highest proportion of area reduction at 62.63%. These findings highlight the effectiveness of using oriented bounding boxes in mitigating the challenges posed by overlapping objects and achieving a more accurate representation of the wheat head.

**Table 3:** Average area comparison of horizontal and oriented bounding box for wheat heads in RGWHD: Quantitative comparison of the difference in the area of wheat ears between oriented and horizontal frames under the same conditions.

Category	Average area(HBB)	Average area(OBB)	Area reduction (HBB—OBB)/HBB
arvalis_1	4442.3	1961.4	55.85%
arvalis_2	5918.8	2212.1	62.63%
arvalis_3	9851.0	5568.7	43.47%
ethz_1	3334.5	2315.0	30.57%
inrae_1	14,867.3	6164.4	58.54%
rres_1	6140.4	3816.4	37.85%
usask_1	12,790.2	5412.4	57.68%

## Methods

### Network architecture

**Figure 3:**
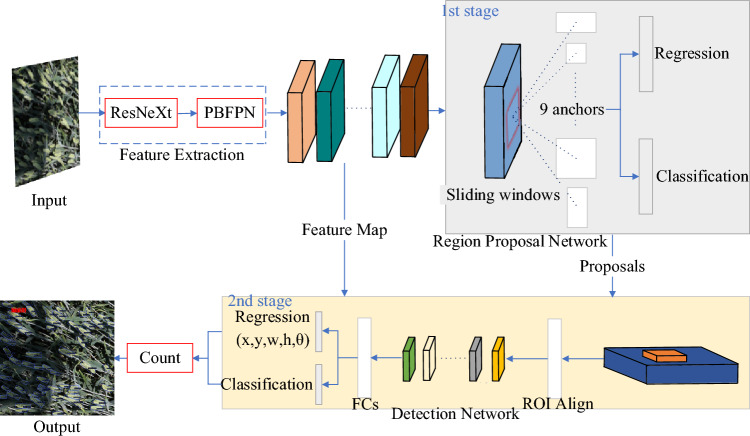
Architecture of the Oriented Feature Pyramid Network (OFPN): Leveraging ResNeXt as the backbone, PBFPN as the feature fusion module, and Soft-NMS for proposal bounding box filtering.

In light of the challenging field conditions for wheat head detection, this study adopts the two-stage high-precision algorithm Faster R-CNN as its foundation. Figure 3 depicts the oriented feature pyramid network (OFPN) built upon Faster R-CNN. The OFPN comprises three interconnected components designed to detect oriented wheat heads within an image: Feature Extraction Network, Region Proposal Network, and Detection Network. The feature extraction network performs as the feature extractor, utilizing a convolutional neural network (backbone) to produce multi-scale features from the input image. To improve the representation of small and densely-packed wheat heads, a feature fusion module called PBFPN has been integrated to merge features from various levels. The region proposal network generates oriented proposals employing a regression branch, while a classification branch determines whether the proposals represent foreground objects. For the final classification and refinement of proposal positions, the detection network applies an oriented R-CNN head.

### Orientation box definition

**Figure 4:**
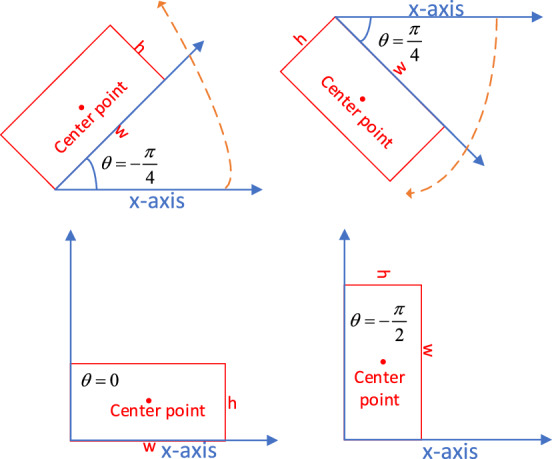
Oriented bounding box representation with long-side definition: Use the long side as the width of the bounding box.

In practice, an oriented bounding box is visually depicted as a slimmer horizontal bounding box that rotates either clockwise or counterclockwise around its center. Along with the corner coordinates, a rotation angle is provided to represent the extent of rotation. Consequently, detecting rotated objects can be achieved through parametric regression of oriented bounding boxes. There are two main methods for representing oriented bounding boxes: the five-parameter method, which utilizes an explicit rotation angle, and the eight-parameter method, which employs the coordinates of the four vertices of a quadrilateral as implicit rotation parameters. This study adopts the five-parameter method with the long-side definition, as illustrated in Fig. 4. In this method, the center coordinates of the oriented bounding box are denoted as x and y, while the width and height are represented by w and h, respectively, with w being greater than h. The rotation angle $\theta$ is determined by the long side (width) and the x-axis, with clockwise being considered the positive direction, and the angle range is specified as $[ - {\pi \mathord{\left/ {\vphantom {\pi 2}} \right. \kern-0pt} 2},{{{\kern 1pt} \pi } \mathord{\left/ {\vphantom {{{\kern 1pt} \pi } 2}} \right. \kern-0pt} 2})$.

### Backbone

In this study, the ResNeXt was chosen as the backbone due to its powerful and efficient design^33^ ResNeXt introduced innovative techniques such as group convolution and the cardinality block. Group convolution employs independent kernels for each group of input channels, enabling parallel computation and reducing computational complexity. The cardinality block aggregates a set of transformations with the same topology, increasing parallel pathways within each block. These parallel pathways allow ResNeXt to learn more diverse and complex features. The key difference between ResNet and ResNeXt lies in the structure of their repeatable residual blocks, as illustrated in Table 4.

**Table 4:** Block structure of ResNet and ResNeXt (cardinality = 32). ResNeXt incorporates a group convolution with a group size of 32 in the repeatable residual block structure.

Stage	ResNet-50	ResNeXt-50(32 × 4d)
conv1	7 × 7, 64, stride 2	7 × 7, 64, stride 2
conv2	3 × 3 max pool, stride 2	3 × 3 max pool, stride 2
	$$\left[\begin{array}{c}\begin{array}{cc}1\times 1,& 64\\ 3\times 3,& 64\end{array}\\ \begin{array}{cc} 1\times 1,& 256\end{array}\end{array} \right]\times 3$$	$$\left[ {\begin{array}{*{20}c} {1 \times 1,128} \\ {3 \times 3,128} \\ {1 \times 1,256} \\ \end{array} , C = 32 } \right] \times 3$$
conv3	$$\left[\begin{array}{c}\begin{array}{cc}1\times 1,& 128\\ 3\times 3,& 128\end{array}\\ \begin{array}{cc}1\times 1,& 512\end{array}\end{array} \right]\times 4$$	$$\left[\begin{array}{c}\begin{array}{cc}1\times 1,& 256\\ 3\times 3,& 256\end{array}\\ \begin{array}{cc}1\times 1,& 512\end{array}\end{array}, C=32 \right]\times 4$$
Conv4	$$\left[\begin{array}{c}\begin{array}{cc}1\times 1,& 256\\ 3\times 3,& 256\end{array}\\ \begin{array}{cc} 1\times 1,& 1024\end{array}\end{array} \right]\times 6$$	$$\left[\begin{array}{c}\begin{array}{cc}1\times 1,& 512\\ 3\times 3,& 512\end{array}\\ \begin{array}{cc} 1\times 1,& 1024\end{array}\end{array}, C=32\right]\times 6$$
Conv5	$$\left[\begin{array}{c}\begin{array}{cc}1\times 1,& 512\\ 3\times 3,& 512\end{array}\\ \begin{array}{cc} 1\times 1,& 2048\end{array}\end{array} \right]\times 3$$	$$\left[\begin{array}{c}\begin{array}{cc}1\times 1,& 1024\\ 3\times 3,& 1024\end{array}\\ \begin{array}{cc}1\times 1,& 2048\end{array}\end{array}, C=32\right]\times 6$$

### Multi-scale feature fusion balanced pyramid (PBFPN)

Feature pyramid networks (FPN)^34^ have become a common module in object detection for their ability to detect objects at various scales. By fusing feature maps from different scales, the model can gather more information on the object's position and semantics, thereby significantly enhancing detection accuracy.

In the case of wheat head detection, the uneven distribution and overlap of wheat heads within an image present challenges for extracting and representing wheat heads' features. Consequently, this adversely affects the accuracy and performance of object detection models. To tackle this issue, we propose PBFPN, an enhanced FPN network, which is based on the Path Aggregation Network (PANet) and Balanced Features Pyramid (BFP).

Firstly, PBFPN constructs two parallel network pathways to effectively capture multi-scale features. The top-down pathway gradually upsamples the feature maps extracted by the backbone from high-level to low-level. The bottom-up pathway aggregates features from higher to lower resolutions and combines its own features with the corresponding higher-level features from the bottom-up pathway through a lateral connection. Prior to the final level in the bottom-up pathway, we employ the Atrous Spatial Pyramid Pooling (ASPP) module to expand the receptive field using different dilation rates. This enables the network to develop a better understanding of the wheat heads at various scales, resulting in improved object detection accuracy and robustness.

**Figure 5:**
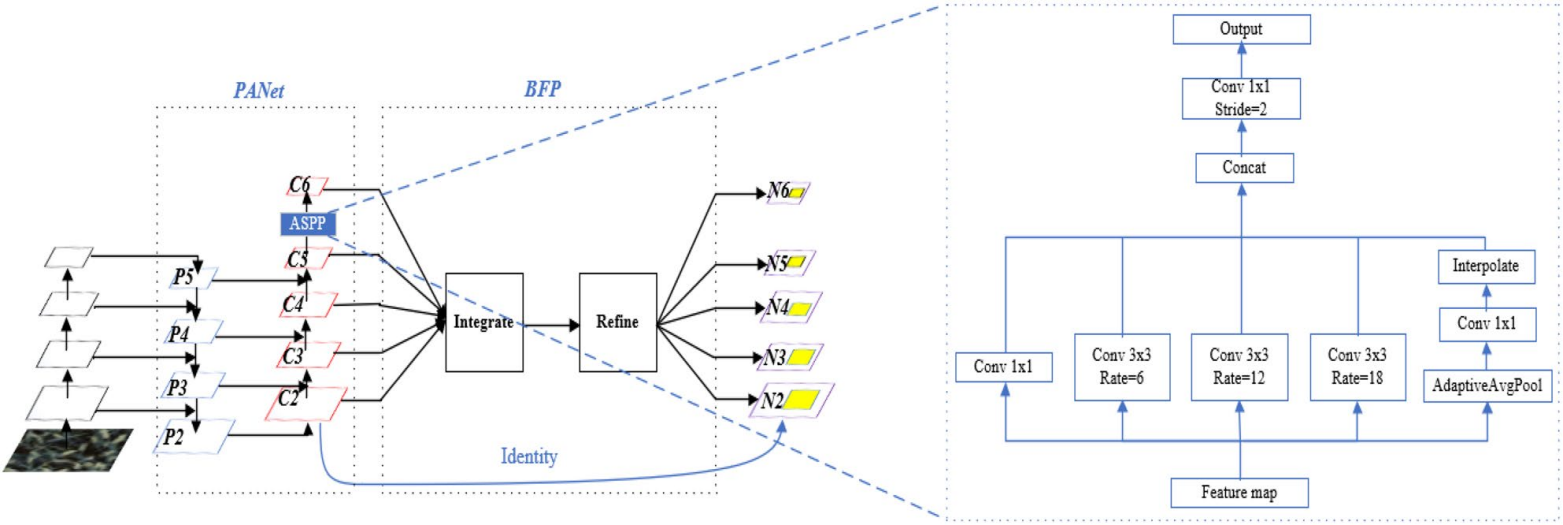
Structure of PBFPN: Incorporating ASPP structure in PANet and combining it with BFP module to enhance object features across multiple scales.

Next, we introduce the Balanced Features Pyramid (BFP) module to tackle the challenges related to fusing features across non-adjacent levels. BFP takes the resulting multi-level features from PANet as inputs, using Interpolation and Max-Pooling to generate feature maps of different scales scaled to medium size, then Integrate the feature maps, and finally re-scaled the resulting features using the same, but reversed, process to enhance the original features. The formula is as follows:

2 $$C = \frac{1}{L}\sum\limits_{{l = l_{\min } }}^{{l_{\max } }} {C_{l} }$$

where $C_{l}$ denotes the resolution of the lth layer of the feature map and $L$ denotes the total number of layers of the feature map, This module ensures a balanced consideration of the importance of multi-level features generated by PANet, resulting in improved performance in detecting objects of different sizes and scales. In summary, we propose a more efficient feature pyramid network named PBFPN, as illustrated in Fig. 5.

### Soft-NMS

The accurate detection of wheat heads in the presence of overlaps presents a significant challenge, particularly when selecting proposal bounding boxes. In dense wheat head detection scenarios, the presence of overlaps, cluttered backgrounds, and variations in wheat appearance may lead to the generation of multiple bounding boxes for the same wheat head. Traditionally, the NMS (Non-Maximum Suppression) algorithm has been employed to select the bounding box with the highest confidence score while suppressing all other proposals. However, this approach may discard potentially valid bounding boxes. To address this limitation, the Soft-NMS algorithm proposed by Bodla^35^, assigns lower scores to overlapping bounding boxes instead of directly eliminating them. By reducing the scores of overlapping proposals, Soft-NMS retains more bounding boxes, allowing for better coverage of objects and minimizing the risk of discarding valuable detections. The degree of suppression increases as the Intersection over Union (IoU) of the proposal bounding boxes with the highest score increases, allowing for a more nuanced selection process in object detection.

$B$ represents the initial detection boxes, $S$ is the set of scores for each detection box, and $D$ corresponds to the set of final detection boxes. $N_{t}$ is the IoU threshold. $b_{m}$ signifies the prediction box with the highest score among all prediction boxes.

The NMS algorithm employs the following function to re-score the neighbor proposal of the detection M with the highest score:

3 $$s_{i} = \left\{ {\begin{array}{*{20}c} {s_{i} ,} & {iou(M,b_{i} ) < N_{t} } \\ {0,} & {iou(M,b_{i} ) \ge N_{t} } \\ \end{array} } \right.$$

Soft-NMS uses a Gaussian function to reduce the confidence scores of the overlapped boxes as follows:

4 $$s_{i} = \left\{ {\begin{array}{*{20}c} {s_{i} ,} & {iou(M,b_{i} ) < N_{t} } \\ {s_{i} (1 - iou(M,b_{i} )),} & {iou(M,b_{i} ) \ge N_{t} } \\ \end{array} } \right.$$

5 $$s_{i} = s_{i} e^{{ - \frac{{iou(M,b_{i} )^{2} }}{\partial }}} ,\forall b_{i} \notin D$$

The equation illustrates that the efficacy of the penalty function depends on the IoU of the prediction bounding boxes. When the intersection ratio is lower than the threshold, the penalty function remains inactive. However, if the intersection ratio exceeds the threshold, the penalty function diminishes the confidence score of the corresponding prediction bounding box.

### Loss

During the RPN phase, positive and negative proposals are represented by 1 and 0, respectively. A positive proposal must meet either of the following conditions: (i) an anchor has an Intersection over Union (IoU) exceeding 0.7 with any ground-truth bounding box, or (ii) an anchor has the highest IoU with a ground-truth bounding box, and the IoU is greater than 0.3. Negative proposals are anchors with an IoU value less than 0.3 in relation to the ground-truth bounding box. Invalid samples, which are neither positive nor negative, are ignored during the training process. In the second stage for Region of Interest (ROI), a proposal is considered positive if its IoU with the true bounding box is greater than 0.5, and negative if its IoU is less than 0.5. The multi-task loss function for each proposal is then defined as:

6 $$F(p,t,v,v^{*} ) = \frac{1}{N}\sum\limits_{n = 1}^{N} {F_{cls} \left( {p_{n} ,t_{n} } \right)} + \lambda \frac{1}{N}\sum\limits_{n = 1}^{N} {F_{reg} \left( {v_{n} ,v_{n}^{*} } \right)}$$

where, $N$ is the number of proposal bounding boxes, $F_{cls}$ is the cross entropy loss for the classification task. $p_{n}$ is the prediction probability generated by the softmax function, indicating whether the anchor is wheat head or background. The variable $t_{n}$ denotes the anchor’s class label, which can take values of either 0 or 1.

7 $$F_{cls} \left( {p_{n} ,t_{n} } \right) = - \log p_{n}$$

${F}_{reg}$ is the ${smooth}_{{L}_{1}}$ loss for the localization regression task. The trade-off between two terms is controlled by the balancing parameter $\lambda$.

8 $$F_{reg} \left( {v_{n} ,v_{n}^{*} } \right) = \sum\limits_{{i \in \{ x,y,w,h,\theta \} }} {smooth_{{L_{1} }} (v_{i} - v_{i}^{*} )}$$

9 $$smooth_{{L_{1} }} (x) = \left\{ {\begin{array}{*{20}c} {0.5x^{2} } \\ {|x| - 0.5} \\ \end{array} } \right.{\kern 1pt} {\kern 1pt} {\kern 1pt} {\kern 1pt} {\kern 1pt} {\kern 1pt} {\kern 1pt} {\kern 1pt} {\kern 1pt} {\kern 1pt} {\kern 1pt} {\kern 1pt} {\kern 1pt} {\kern 1pt} \begin{array}{*{20}c} {if\;{\kern 1pt} \left| x \right| < 1} \\ {otherwise} \\ \end{array}$$

where, ${\text{v}}$ = (${{\text{v}}}_{{\text{x}}}$, ${{\text{v}}}_{{\text{y}}}$, ${{\text{v}}}_{{\text{w}}}$, ${{\text{v}}}_{{\text{h}}}$, ${{\text{v}}}_{\uptheta }$) is a ground truth bounding box regression tuple containing the coordinates of the center point, the width and height, and the angle of rotation. ${{\text{v}}}^{*}$ = (${{\text{v}}}_{{\text{x}}}^{*}$, ${{\text{v}}}_{{\text{y}}}^{*}$, ${{\text{v}}}_{{\text{w}}}^{*}$, ${{\text{v}}}_{{\text{h}}}^{*}$, ${{\text{v}}}_{\uptheta }^{*}$) is the predicted tuple for the target.

### Experiments

We conducted comprehensive experiments on the RGWHD dataset to evaluate the performance of the proposed model, comparing it with state-of-the-art (SOTA) models.It is worth noting that the resolution of the training images is 1024 × 1024.

### Experimental setup

All experiments are conducted on the deep learning framework PyTorch with Python 3.7 as the programming language. All models were trained for 30 epochs. The initial learning rate is set to 0.005 and then decreased at epochs 8. The models were trained and tested on a desktop computer with an Intel Core i7-8700 CPU @3. 20 GHz × 6, 24 GB of RAM and a GeForce GTX 1080Ti GPU, running Ubuntu 18.04.5 LTS.

### Evaluation metrics

In this study, we evaluated the model’s accuracy in terms of the mean Average Precision (mAP) metric, which combines four key measures: True Positive (TP), False Positive (FP), False Negative (FN), and True Negative (TN). True Positive (TP) indicates the correct prediction of a wheat head. False Positive (FP) refers to the incorrect prediction of a wheat head that is not present in the image. False Negative (FN) represents the missed prediction of an actual wheat head. True Negative (TN) represents the correct prediction of an object that is not present in the image.

The aforementioned measures are widely utilized in the computation of evaluation metrics, including precision, recall, Average Precision (AP), and mAP, which offer valuable insights into the performance of object detection models.

Intersection over Union (IoU) is a crucial metric that quantifies the extent of overlap between the predicted bounding box and the ground truth bounding box. It is computed as the ratio of the intersection area to the union area of the two bounding boxes. In object detection tasks, a specific IoU threshold is commonly defined to to determine TP and FP.

10 $$IoU = \frac{{S_{ \cap } }}{{S_{ \cup } }}$$

Precision: the percentage of true positives detected by the model among all the objects predicted.

11 $$P = \frac{TP}{{TP + FP}}$$

Recall: the percentage of true positives detected by the model among all the ground truth objects.

12 $$R = \frac{TP}{{TP + FN}}$$

AP (average precision): the average precision at different recall levels. It is computed by integrating the precision values along the Precision-Recall (PR) curve.

13 $$AP = \int_{0}^{1} {P\left( R \right)d_{R} }$$

mAP: the average of AP across all categories.

14 $$mAP = \frac{{\sum\nolimits_{i = 1}^{N} {AP_{i} } }}{N}$$

### Ablation study

We conducted ablation experiments to assess the impact of individual components within the proposed OFPN. The modules examined included ResNeXt, PBFPN, and Soft-NMS. The base model was constructed using the Rotated Faster RCNN with ResNet50 as its backbone. Various configurations were then applied for training and validation on the RGWHD dataset. The experimental results successfully demonstrated the effectiveness of combining these three modules in enhancing the overall performance of OFPN. Table 5 presents the results, indicating a notable 4.14% improvement in detection accuracy (mAP) compared to the base model.

**Table 5:** Ablation study results of the proposed model OFPN on the RGWHD dataset, where bold numbers represent the best result.

Method	ResNeXt	PBFPN	Soft-NMS	arvalis_1	arvalis_2	arvalis_3	ethz_1	inrae_1	rres_1	usask_1	mAP/%	Recall/%
base				0.894	0.657	0.792	0.808	0.872	0.898	0.793	81.63	88.57
	√			0.896	0.699	0.819	0.805	0.878	0. 898	0.795	82.62	89.68
		√		0.895	0.736	0.863	0.808	0.887	0. 902	0.795	84.09	90.45
			√	0.892	0.724	0.827	0.878	0.859	0. 899	0.855	84.78	91.26
OFPN	√	√	√	0.895	0.755	0.851	0.886	0.873	0. 899	0.865	**85.77**	**92.54**

### Wheat heads detection results

**Figure 6:**
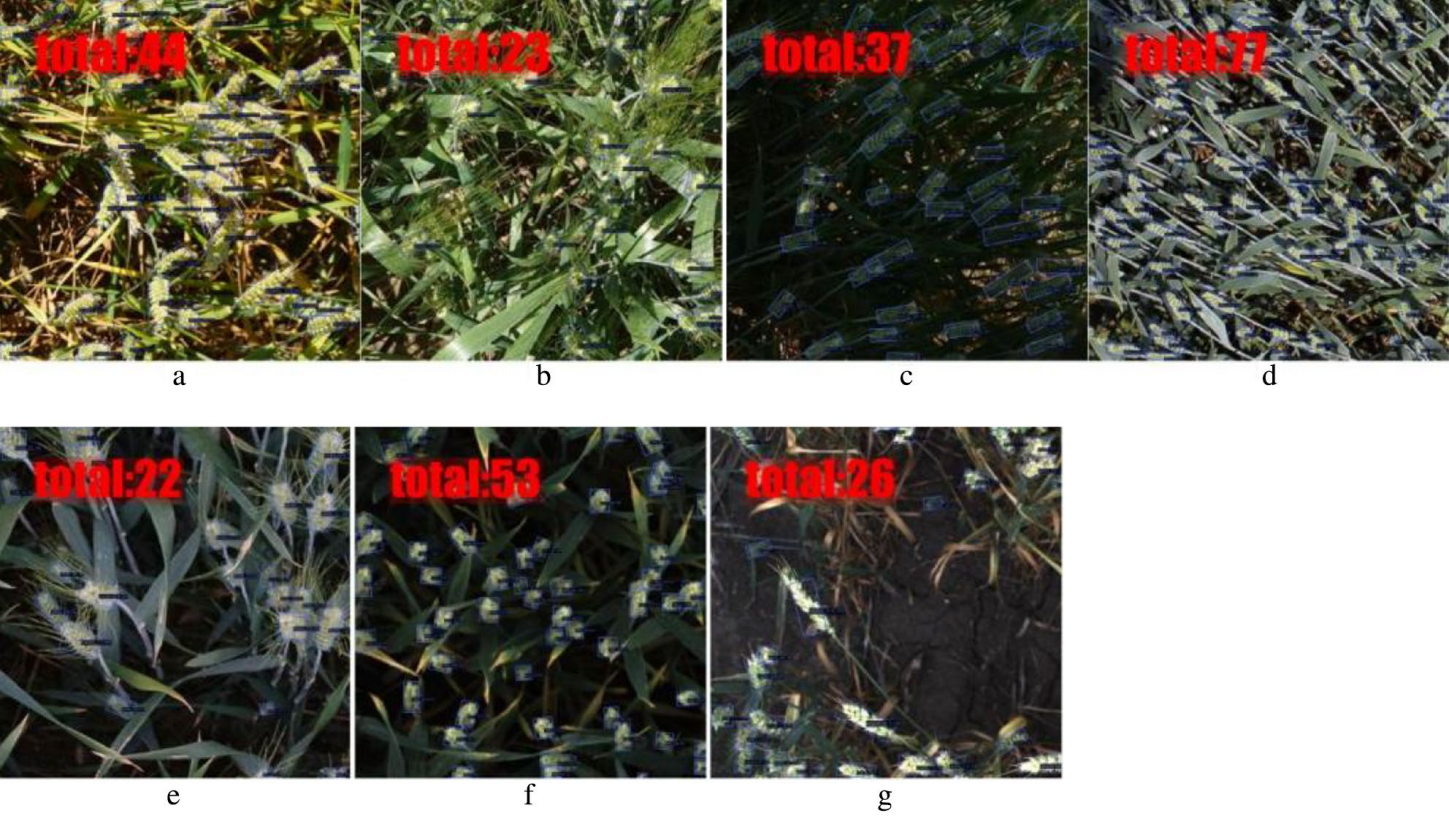
Wheat heads detection results with OFPN on RGWHD: (**a**) arvalis_1, (**b**) arvalis_2, (**c**) arvalis_3, (**d**) ethz_1, (**e**) inrae_1, (**f**) rres_1, (**g**) usask_1.

We conducted experiments on the RGWHD dataset and evaluated the performance of Oriented Feature Pyramid Network (OFPN) in terms of mAP. For each catogory, the dataset is split into training, validation, and testing sets in a ratio of 7:1:2. Figure 6 presents the visualization of prediction results for the seven wheat categories. The proposed model accurately predicts the oriented bounding boxes for the wheat heads in each test image, while also providing additional information including the total count of detected heads and the classification probability for each detection.

**Figure 7:**
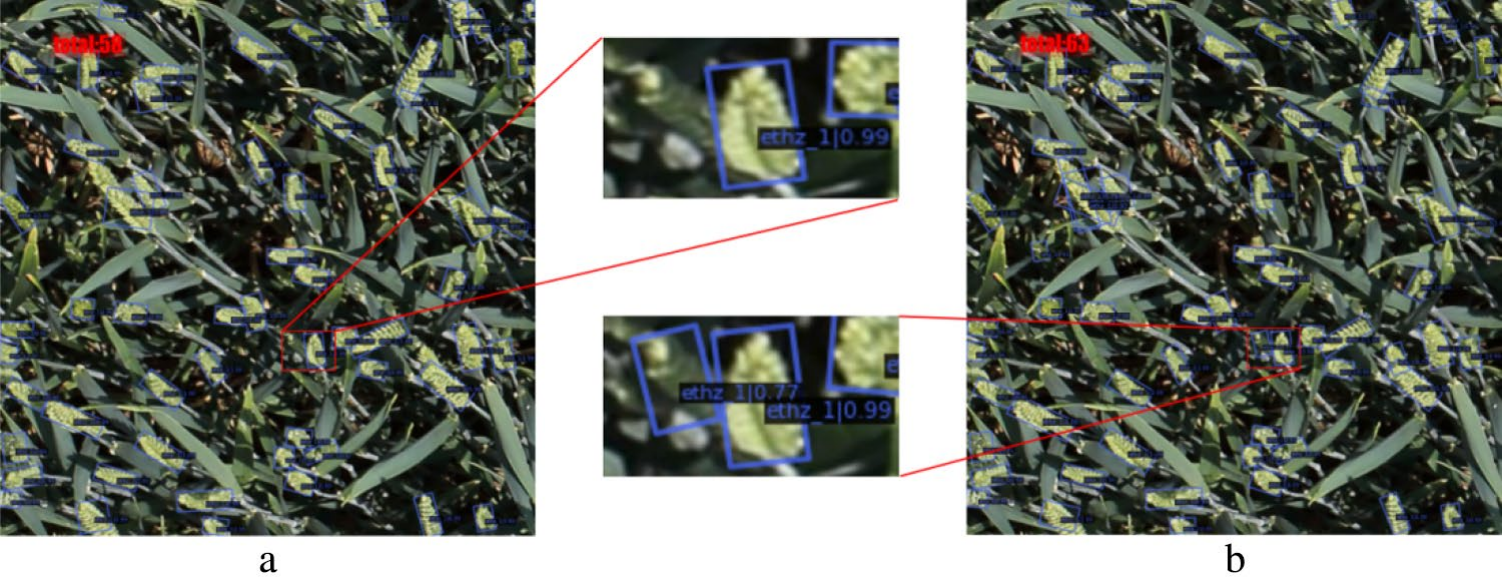
Enlarged detail of the same prediction result image: (**a**) Faster R-CNN, (**b**) OFPN.

The size of the RGWHD image is 1024 × 1024 pixels, which makes the bounding boxes in Fig. 7 difficult to discern. In order to provide a more detailed demonstration of the oriented object detection results, we evaluated two similar models based on R-CNN. The zoomed details of the same image can be seen in Fig. 7. Using the same settings for the threshold of IoU and bounding box score, the proposed OFPN detected an additional 5 small and overlapping wheat heads compared to Faster RCNN.

**Figure 8:**
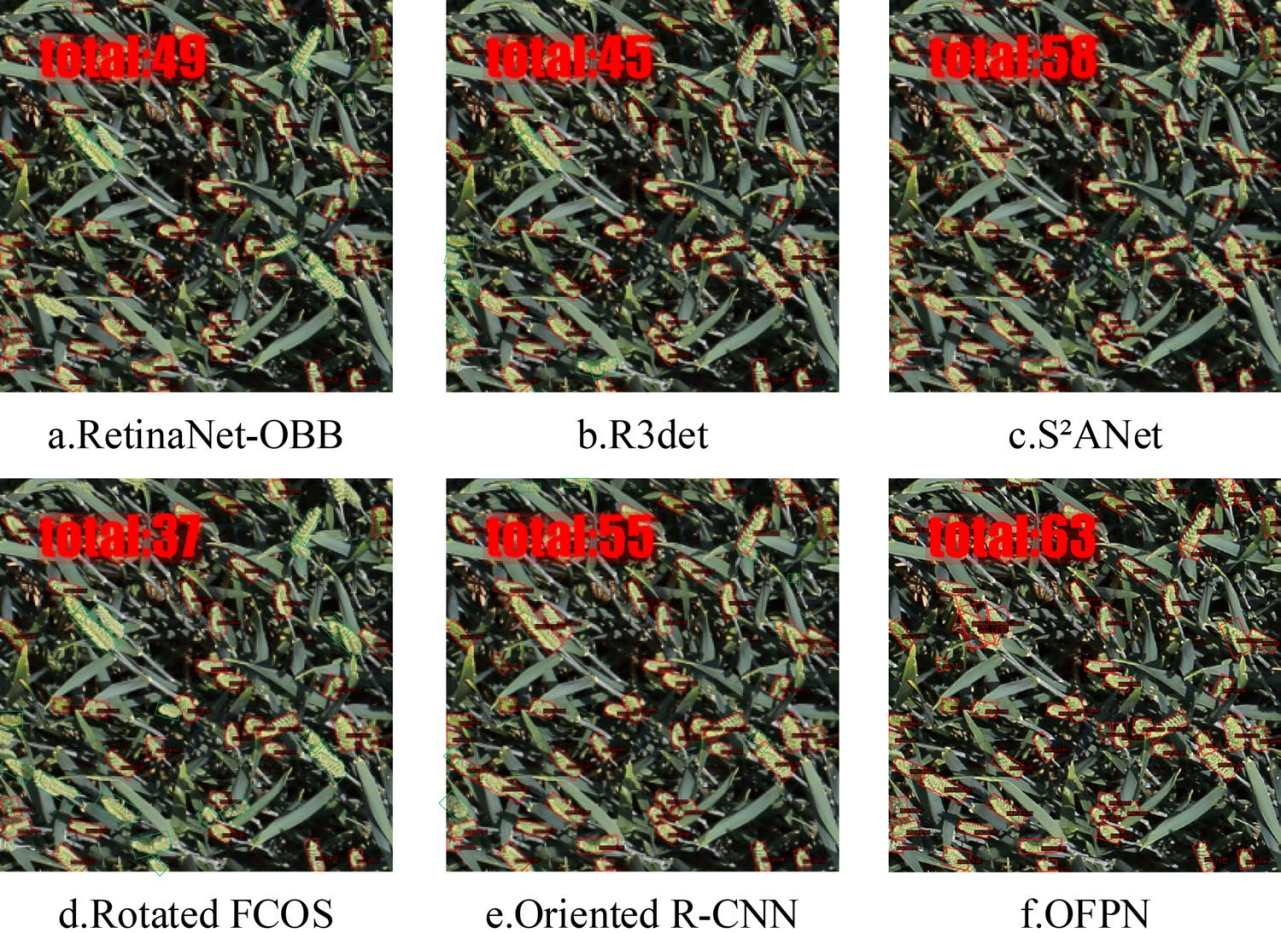
Comparison of different model predictions: using the green box to enclose targets missed by the model.

**Figure 9:**
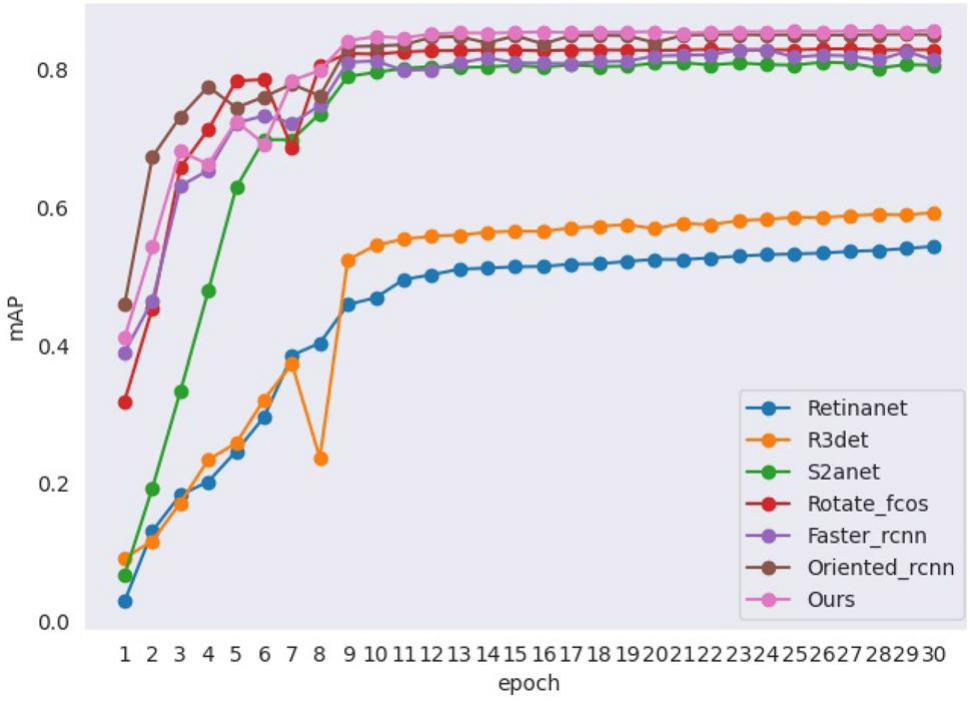
mAP during training for all comparative models.

We conduct a comparative analysis of our Oriented Feature Pyramid Network (OFPN) with six state-of-the-arts oriented object detection models on the RGWHD dataset. These models include both one-stage detection models, such as Rotated RetinaNet^36^, R3Det^37^, S2ANet^38^, Rotated FCOS, as well as two-stage detection models including Faster RCNN-OBB^10^, Oriented RCNN^23^. The results, which are presented in Table 6 and Fig. 8, demonstrate that the two-stage detection models perform better compared to the one-stage detection models. Among the seven categories of wheat, the detection tasks for varieties arvalis_2, arvalis_3, usask_1 are more challenging for all the models we compared. However, our model exhibits the best performance compared to all the other state-of-the-art models. For the rest wheat varieties, OFPN achieves almost equivalent accuracy with the best model Faster RCNN. Furthermore, Fig. 9 shows the validation mAP of all the comparative models during the training process.

**Table 6:** Comparison results with state-of-the-art oriented object detection models on the RGWHD dataset, where bold numbers represent the best result.

Method	arvalis_1	arvalis_2	arvalis_3	ethz_1	inrae_1	rres_1	usask_1	mAP/%	Recall/%	GFlOPs	Parameters/M
One-Stage											
RetinaNet-OBB	0.747	0.519	0.435	0.759	0.487	0.595	0.264	54.38	86.07	212.29	36.25
R3Det	0.854	0.458	0.209	0.793	0.796	0.641	0.398	59.27	86.79	331.72	41.72
S^2^ANet	0.892	0.699	0.750	0.804	0.826	0.894	0.783	80.68	88.52	197.62	38.6
Rotated FCOS	0.895	0.749	0.799	0.804	0.878	0.896	0.782	82.91	89.68	206.5	31.9
Two-Stage											
Faster RCNN-OBB	0.894	0.657	0.792	0.808	0.872	0.898	0.793	81.63	88.57	211.29	41.13
Oriented R-CNN	0.898	0.742	0.843	0.888	0.892	0.899	0.795	85.08	90.43	211.42	41.13
OFPN(Ours)	0.895	0.755	0.851	0.886	0.873	0.899	0.865	**85.77**	**92.54**	242.87	46.65

### Classification results

**Figure 10:**
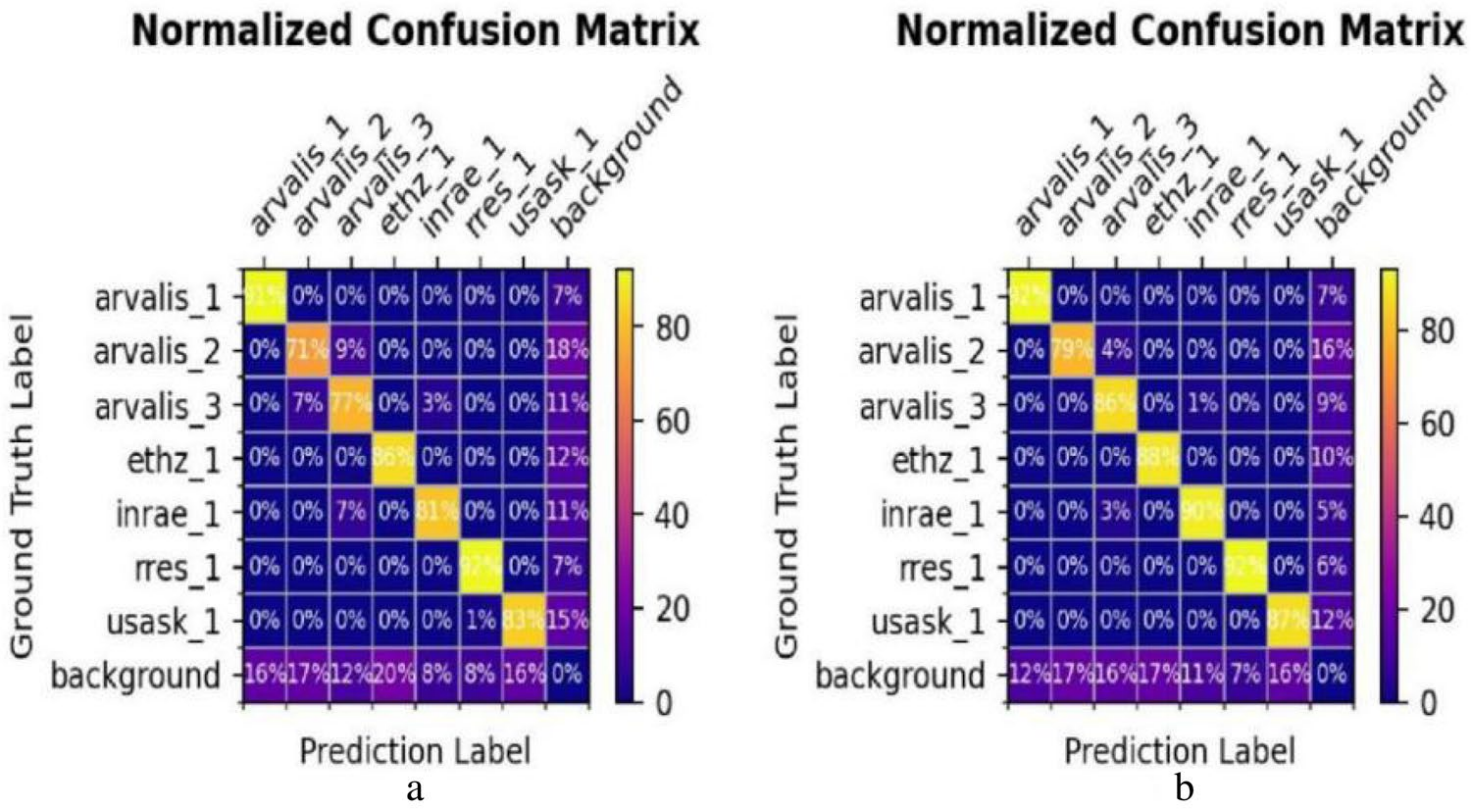
Comparison of the classification results in confusion matrix: (**a**) Faster R-CNN, (**b**) OFPN.

To assess the model's object recognition capabilities, we compared the performance of the proposed OFPN with the high-performance Faster R-CNN using their respective confusion matrices. Figure 9 illustrates the prediction probabilities for different wheat categories in the test set, with rows representing actual classes and columns representing predicted classes. The analysis of Fig. 10 reveals that Faster R-CNN tends to misclassify the arvalis_2 and arvalis_3 categories. In contrast, OFPN significantly enhances the classification capability for all wheat categories. These results indicate that our proposed model effectively extracts aggregated features from the images, thereby mitigating the influence of factors such as an appearance on wheat head classification.

### Wheat head counting

To evaluate the counting performance of wheat head detection models, we established a threshold of 0.7 for the confidence score of prediction proposals across all comparative models. Among the 140 images in the test set, a total of 5074 ground truth bounding boxes were identified. Table 7 presents the results, indicating that OFPN achieved a wheat head counting accuracy of 93.97%, surpassing Faster R-CNN by 3.13%. It is worth noting that both Faster R-CNN and OFPN exhibited subpar counting performance for wheat arvalis_2. This can be attributed to the challenging lighting conditions under which the arvalis_2 images were captured, making it difficult to distinguish wheat heads from the background.

**Table 7:** Wheat head counting results on the RGWHD dataset, the bold fonts indicate the best results.

Method	arvalis_1	arvalis_2	arvalis_3	ethz_1	inrae_1	rres_1	usask_1	Total
Manual counting	824	389	506	1469	371	901	614	5074
Faster R-CNN	795	**319**	436	1301	352	846	560	4609
Accuracy	96.48%	**82.01%**	86.17%	88.56%	94.88%	93.90%	91.21%	90.84%
OFPN(Ours)	**802**	317	**483**	**1348**	**383**	**851**	**584**	**4768**
Accuracy	**97.33%**	81.49%	**95.45%**	**91.76%**	**96.77%**	**94.45%**	**95.11%**	**93.97%**

## Conclusion

Wheat heads detection and counting are significant for various purposes, including visual object detection, wheat yield estimation and planting management. Similar to ships in aerial images, wheat heads often appear with arbitrary orientations in field images. Motivated by the oriented object detection in DOTA dataset, we propose a new Rotated Global Wheat Head Dataset (RGWHD). We also present an Oriented Feature Pyramid Network (OFPN) for the detection of small and dense wheat heads. OFPN enhances the representation of small wheat heads through the utilization of a multi-scale feature fusion network known as PBFPN. Furthermore, it handles the detection of dense wheat heads with a Soft-NMS by assigning lower scores to overlapping boxes. OFPN performs well in the tasks of oriented wheat heads detection, category recognition and wheat heads number prediction. Considering the extensive distribution and diverse varieties of wheat, as well as the challenges associated with dataset collection, our future research will focus on wheat detection models under weakly supervised conditions.

## Data Availability

The datasets generated and analysed during the current study are available in RGWHD (https://pan.baidu.com/s/1Fy3HpIfAeQhRef_ZuKu4iw) and the extraction code is vbiy. The datasets generated during and/or analyzed during the current study areavailable from the corresponding author on reasonable request. The scripts to run all experiments are publicly available through our GitHub page https://github.com/cwr0821/OFPN.
